# Brain-Computer Interfaces Systems for Upper and Lower Limb Rehabilitation: A Systematic Review

**DOI:** 10.3390/s21134312

**Published:** 2021-06-24

**Authors:** Daniela Camargo-Vargas, Mauro Callejas-Cuervo, Stefano Mazzoleni

**Affiliations:** 1 Software Research Group, Universidad Pedagógica y Tecnológica de Colombia, Tunja 150002, Colombia; daniela.camargo01@uptc.edu.co; 2 School of Computer Science, Universidad Pedagógica y Tecnológica de Colombia, Tunja 150002, Colombia; 3 Department of Electrical and Information Engineering, Politecnico di Bari, 70126 Bari, Italy; stefano.mazzoleni@poliba.it

**Keywords:** brain computer interfaces (BCIs), electroencephalography (EEG), rehabilitation, upper limb, lower limb, virtual reality

## Abstract

In recent years, various studies have demonstrated the potential of electroencephalographic (EEG) signals for the development of brain-computer interfaces (BCIs) in the rehabilitation of human limbs. This article is a systematic review of the state of the art and opportunities in the development of BCIs for the rehabilitation of upper and lower limbs of the human body. The systematic review was conducted in databases considering using EEG signals, interface proposals to rehabilitate upper/lower limbs using motor intention or movement assistance and utilizing virtual environments in feedback. Studies that did not specify which processing system was used were excluded. Analyses of the design processing or reviews were excluded as well. It was identified that 11 corresponded to applications to rehabilitate upper limbs, six to lower limbs, and one to both. Likewise, six combined visual/auditory feedback, two haptic/visual, and two visual/auditory/haptic. In addition, four had fully immersive virtual reality (VR), three semi-immersive VR, and 11 non-immersive VR. In summary, the studies have demonstrated that using EEG signals, and user feedback offer benefits including cost, effectiveness, better training, user motivation and there is a need to continue developing interfaces that are accessible to users, and that integrate feedback techniques.

## 1. Introduction

Brain-computer interfaces (BCIs) represent a broad field of research and development from the last few decades. Scientists from all over the world have worked to acquire a deep understanding of BCIs, resulting in rapid and considerable progress in systems, development, and brainwave processing techniques including non-invasive methods such as electroencephalography (EEG) [1]. Most relevantly, useful and novel applications developed in this domain have contributed to the evolution of technology in healthcare [2]; it is clearly evidenced that using a BCI system plays an efficient and “natural” role in the attempt to provide assistance and preventive care to people with neurological disorders [3,4].

The fields where BCIs can be applied are quite promising and diverse. In fact, it is becoming an innovative neurological technology that successfully allows the restoration and improvement of people’s motor and communication abilities [5,6]. According to [7,8,9,10,11,12], its application fields can be divided into communication and control, medical applications, training and education, games and entertainment, monitoring, prevention, detection and diagnosis, intelligent environments, neuromarketing, advertising, security, and authentication. However, this review focuses specifically on examining the applications proposed exclusively as support in rehabilitation processes of the upper and lower limbs of the human body.

For people with partial or total limitation of movement in the upper and lower limbs, using a BCI and classifying an EEG recorded over the sensorimotor cortex in real time gives the possibility of understanding psychological and motor parameters and intentions that allow the reestablishment of communication with the environment [13,14,15]. In short, BCI technology provides direct communication between the brain and an external device, which can assist with numerous diseases [16] such as epilepsy [17], Alzheimer’s disease [18], traumatic brain injuries [19], strokes [20,21], neurological diseases [22], multiple sclerosis [23], and Parkinson’s disease [24]. Indeed, this problem demands innovative advances that counteract the varied impacts that humanity has on a social level, with the economic situation and life of each patient and his or her family coming into play [25].

With the goal of identifying advances and opportunities for improvement in the rehabilitation of upper and lower limbs in the human body, this review includes studies published between 2011 and 2020. In line with the goal of the review, for the included proposals, the devices used for EEG signal acquisition are described, as well as the processing methods within the system, and the BCI applications that support rehabilitation processes in the upper and lower limbs. On the other hand, it was observed that most studies involved BCI systems focused on the upper limbs, and the use of EEG signals and user feedback showed improvements in BCI systems. The objective of this review is to identify the contributions that have been made so far as well as describing opportunities for improvement and limitations that should be taken into account in order to guide future proposals focused on supporting rehabilitation processes of limbs of the human body based on the treatment of EEG signals and user feedback.

## 2. Materials and Methods

This section describes the process and criteria taken into account to identify, select, and evaluate relevant papers, as well as to collect and analyse the data from the studies of the articles included in the document review, according to the Preferred Reporting Items for Systematic Reviews and Meta-Analyses (PRISMA) method [26,27,28].

### 2.1. Eligibility Criteria

The eligibility criteria taken into consideration for the inclusion of studies in this review were: (i) publications in English language and (ii) publications with full text availability. A relevant element in this review is that the included studies have described BCI applications in body limb rehabilitation processes and included virtual reality (VR) environments as part of the feedback.

### 2.2. Search Strategy

A search was carried out on the 23rd of February, 2021, using the PubMed, IEEE Xplore, Scopus, and Web of Science databases. The keywords used in the search were obtained according to the inclusion criteria, whose search terms were classified as follows: (i) medical aspects: “rehabilitation”; (ii) use of electroencephalographic signals: “electroencephalography”, “EEG”; (iii) body part on which rehabilitation is focused: “upper limb”, “lower limb”, “hand”, “leg”; (iv) design of brain-computer interfaces: “BCI”; (v) use of virtual environments: “virtual” and “augmented”. In the search parameters used in the databases, the OR operator was included for groups among the terms considered synonyms and the AND operator was used to separate groups.

### 2.3. Description of the Selection Process of the Study

The paper selection process included four stages: first, the identification of studies according to the search parameters; second, the application of a filter using the eligibility criteria; third, a screening phase, which filtered the studies, eliminating those that did not fit the research approach according to the inclusion and exclusion criteria in Table 1, and/or those that appear in multiple databases; and finally, an inclusion phase, which allowed the identification of relevant studies to be included in the review.

### 2.4. Data Collection Process

The data collection process for extracting relevant information from the articles was based on an information independent matrix. For each included article, the main characteristics were extracted, as shown in Table 2. In addition, for each proposal, the following items were extracted: the number of electrodes used, the EEG signal acquisition devices used, the EEG signal processing and classification methods used, the operation of the BCI application, and the integrated virtual environment. These processing details are described throughout Section 3.

## 3. Results

This section presents the findings obtained from the literature review, as well as the characteristics of the papers included in the systematic review and the individual results presented in the selected publications.

### 3.1. Selection of the Study

**Figure 1:**
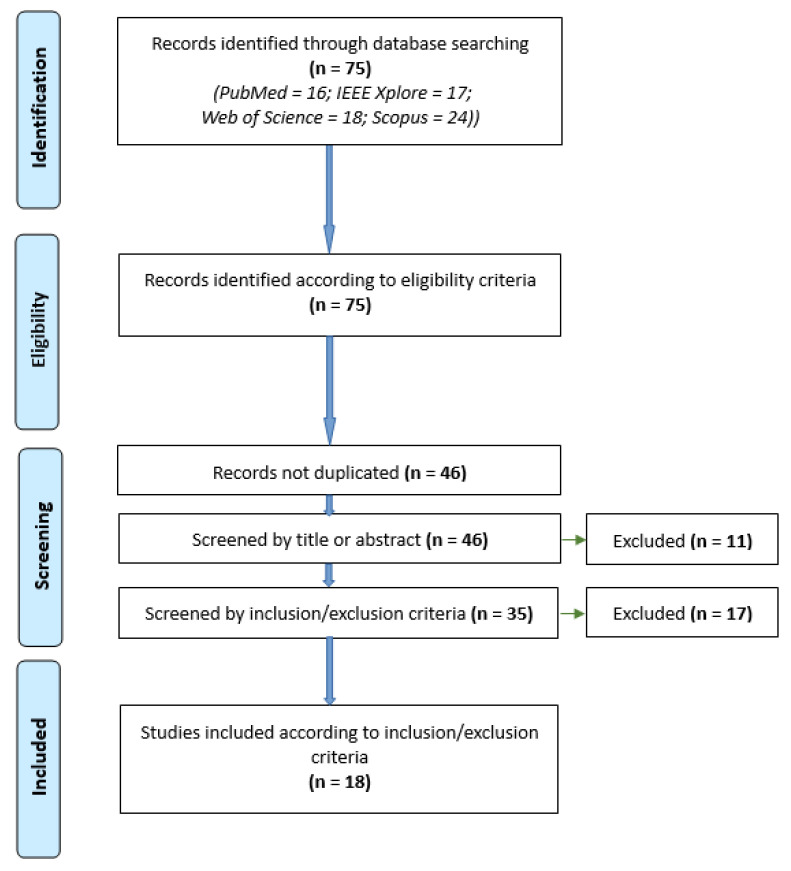
Chart for identifying relevant studies.

The chart for the systematic review is presented in Figure 1. In the first stage, “identification”, the results of which came from documents published between the years of 2003 and 2020. In short, 46 articles were eliminated (18 duplicates and 28 according to exclusion criteria), which meant that 18 studies were included in the review. The 18 studies finally included were published between 2011 and 2020.

### 3.2. General Characteristics of the Study

The main characteristics of the 18 studies included in the review are classified into five groups: (i) limb to be rehabilitated (upper, lower, or both); (ii) purpose of the proposed BCI application (assistance with movement or motor intention); (iii) type of BCI application; (iv) type of feedback to the user; (v) type of virtual reality used for the rehabilitation process.

**Figure 2:**
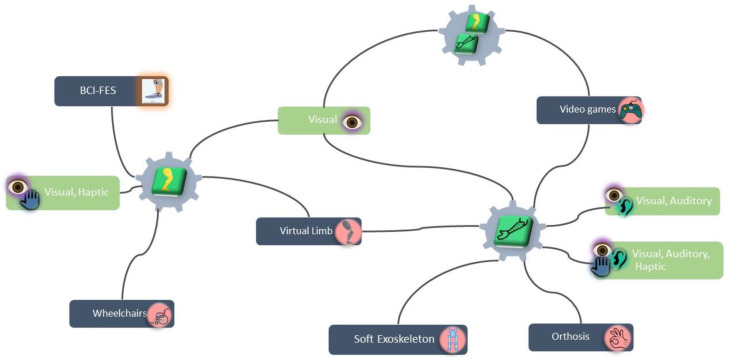
Interconnection between some relevant characteristics of the study.

Figure 2 presents a general map of some relevant characteristics of the study, revealing the focus of the included proposals and the connections between the limb to be rehabilitated (upper, lower, or both), the type of BCI application, and the type of feedback provided to the user.

**Figure 3:**
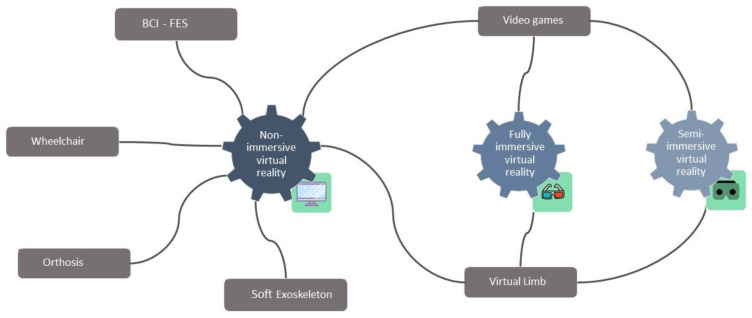
Interconnection between the type of BCI application and the type of virtual reality employed.

In addition, Figure 3 presents a general map of the connections between the type of BCI application and the type of virtual reality used for the rehabilitation process.

#### 3.2.1. Type of Limb Being Rehabilitated

According to the review of the 18 studies included, 11 of them corresponded to BCI systems whose objective was to rehabilitate upper limbs, six focused on rehabilitation of lower limbs, and one was oriented towards rehabilitating both limbs.

#### 3.2.2. Purpose of the BCI Application (Assistance with Movement or Motor Intention)

BCI applications are developed to restore motor function inducing activity-dependent brain plasticity or by providing control over assistive devices that augment or restore movement [47]. Of the 18 studies included in the review, 10 of them presented applications whose purpose was associated with movement intention, i.e., the user’s attempt to move the limb autonomously. In contrast, eight of the studies were focused on applications whose purpose was to assist movement, i.e., the support provided by a device to assist in the movement of the limb, such as neuro-prostheses, mobile robots or wheelchairs [48,49].

#### 3.2.3. Type of BCI Application

In the review process, it was observed that the BCI applications described in the literature by the included works supported rehabilitation processes and were divided into the following categories: eight simulated virtual limbs, five implemented video games, two (11%) were oriented towards the use of wheelchairs, one towards the use of orthoses, one towards integration with functional electrical stimulation (FES), and one focused on the activation of soft exoskeletons.

#### 3.2.4. Type of Feedback Provided to User

Regarding the type of feedback provided to the user, in other words, the different modalities provided by the BCI system (visual, auditory, and haptic [50]) from which the subject perceives sensations, it was shown that eight of the proposed works was based exclusively on visual feedback, six on combined visual and auditory feedback, two on haptic and visual feedback, and two implemented visual, auditory, and haptic feedback.

#### 3.2.5. Type of Virtual Reality Employed

Dividing the 18 proposals according to three types of virtual reality employed, it was observed that four made use of fully immersive virtual reality, i.e., it has realistic simulations, complete with sight and sound, where the user needs to wear appropriate virtual reality glasses or a head-mounted display [51]. In three of the studies, semi-immersive virtual reality was utilized, in other words, the environment is partially virtual, as users perceive a different reality when they focus on a digital image, but they are also connected to their physical environment [52]. Finally, 11 used non-immersive virtual reality, i.e., a computer-generated environment is provided, allowing the user to be fully aware of and maintain control of their physical environment. These environments rely on a computer or video game console and a screen [53,54].

### 3.3. BCI Systems as Support for the Motor Rehabilitation of Human Limbs

**Figure 4:**
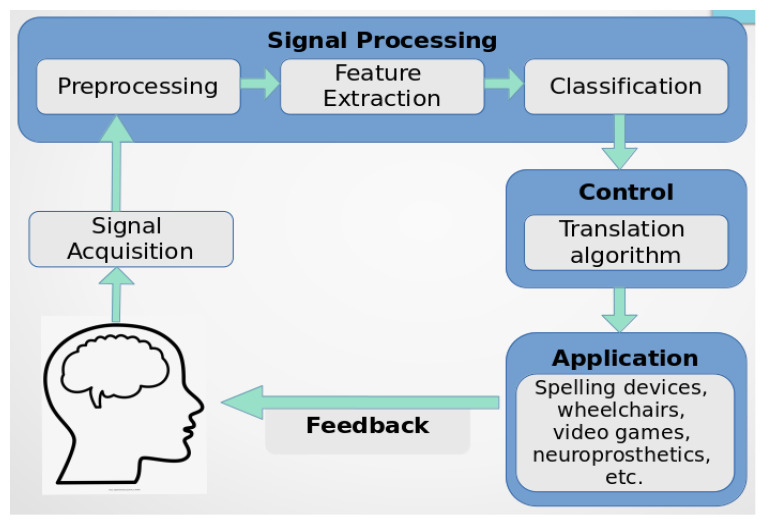
Architecture of a Brain Computer Interface.

A brain-computer interface (BCI), whose architecture is shown in Figure 4, is a system that receives brain signals in order to analyse, transfer, and translate them into commands that are then transmitted to applications that perform particular tasks, thus providing direct communication between the brain and any external device. This avoids the use of normal neuromuscular pathways [9,55,56,57].

In order to know the main details of the architecture of the BCI system proposed in each included article, an extraction of information was carried out as mentioned in Section 2.4. Throughout this section and according to this extraction, for each of the 18 documents analysed, the following are described: the number of electrodes used, signal acquisition, preprocessing, feature extraction, classification, operation of the BCI application, feedback, and the findings given by the authors.

Table 2 presents the main characteristics of each of the 18 studies analysed and identifies how they supported rehabilitation through various categories of applications and feedback. In this sense, several articles focused on upper and lower limb rehabilitation were found in the literature, thus showing strong trends in the use of visual feedback and non-immersive virtual reality.

#### 3.3.1. Processing Techniques and Signal Classification Methods

A brain-computer interface, as presented in Figure 4, consists of the following steps: signal acquisition, preprocessing, feature extraction, classification, translation to commands, application, and feedback [4,9,10].

After the first step, which corresponds to acquiring brain waves, i.e., measuring and removing noise from the recorded brain signal to enhance the relevant information it contains [11], signal processing is performed. In this step, the non-relevant information is removed and the acquired signal is prepared, filtered in such a way that the associated time or frequency information is acquired, the features are extracted and selected, and the dimensionality is reduced in such a way that the requirements for the design of the BCI are obtained in an optimal and efficient way [58,59,60]. According to [4,11,61,62,63], the following signal processing techniques are commonly used:

- Spatial filters: low-pass, high-pass, band-reject, band-pass, common average reference (CAR) or surface Laplacian (SL).
- Temporal filters: discrete Fourier transform (DFT), finite impulse response (FIR), or infinite impulse response (IIR).
- Feature extraction methods: amplitude extraction from spectra of EEG signals, autoregressive parameters or Hjorth parameters, band powers (BP), wavelets, cross-correlation between EEG band powers, frequency representation (FR), power spectral density (PSD), time frequency representation (TFR), short-time Fourier transform (STFT), Wigner-Ville distributions, adaptive Gaussian representations, parametric modeling, time frequency representations with Q-constant frequency decomposition, inverse model and specific techniques used for P300 and VEP such as peak picking (PP), calculation of slow cortical potentials (SCPs), fractal dimension of signals or multifractal spectrum.
- Dimensionality reduction: spatial filters, referencing methods, principal component analysis (PCA), independent component analysis (ICA), common spatial patterns (CSP), common spatial subspace decomposition (CSSD), frequency normalization (Freq-Norm), or genetic algorithms.

**Figure 5:**
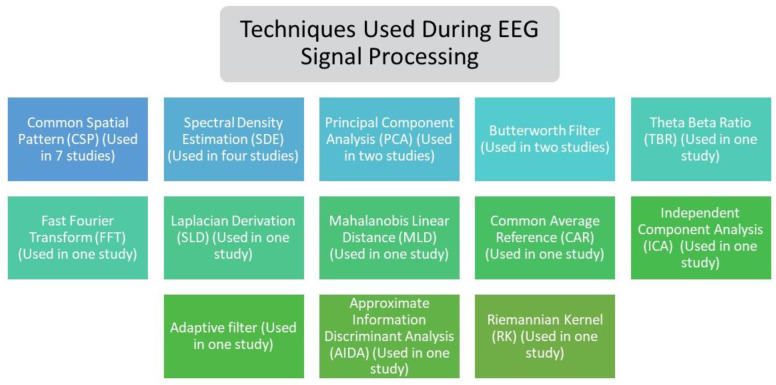
Techniques applied during EEG signal processing of the included proposals.

According to the exploration carried out in each of the studies, the different techniques applied during EEG signal processing presented in Figure 5 were extracted. It was observed that, in terms of feature extraction methods [64], the common spatial patterns (CSP) algorithm is widely used, and spectral density estimation (SDE), a statistical procedure used to estimate the spectral density of a random signal [65], is among the most commonly used techniques within EEG processing. Similarly, it is evident that a wide variety of techniques were employed for feature extraction, filtering, and analysis of practical signals in BCI.

**Figure 6:**
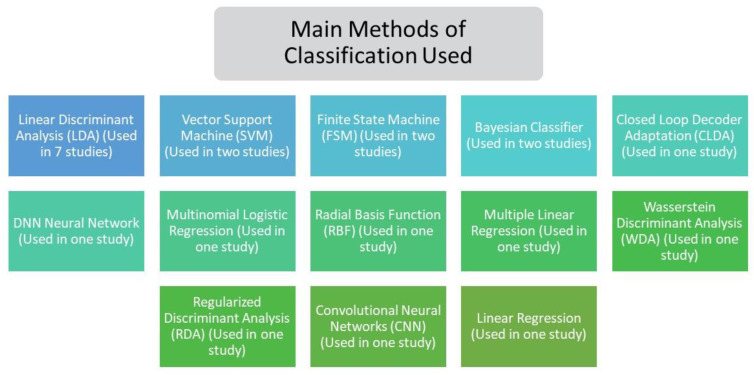
Methods used in the classification and modeling of the control system of the included proposals.

On the other hand, as shown in Figure 6, it was demonstrated that among the main methods used to perform the classification and modeling of the control system, i.e., to categorise that class of feature vectors previously obtained, in order to represent the type of mental task being performed by the user of the BCI system [4], the linear discriminant analysis is the most commonly used. LDA is a nonparametric method since it maximizes between-group variability relative to within-group variability, but it can also be a parametric procedure for classification [66].

Furthermore, the proposal made by [44] was one of the most novel studies included in the review, since it included deep learning (DL) algorithms and convolutional neural networks (CNN). DL is a subfield of machine learning concerned with implementations of multilayer stacks of modules, which learn and compute non-linear input-output mappings and, one of the important aspects is that these layers of features are not designed by humans, they learn from data [67,68]. On the other side, a CNN is a feature extractor, a classifier, and a regressor, which is set up from multiple feature maps. A CNN is designed to process data that come in from multiple arrays and its artificial neurons in each feature map share the same set of weights, but they apply their weights to a different part of the input [67,69].

Additionally, the authors of the proposal indicated that these classifiers performed better than previous architectures, achieving hit rates of up to 78.6% for one of the classes performed. In the study, a motor imagery-BCI system (MI-BCI) was developed with a virtual reality game for the limbs of the human body. The dataset was formed by 1500 EEG recordings, 16 electrodes identified according to the international 10–20 system with a sampling frequency of 160 Hz were used to acquire the EEG signals, a sixth order Butterworth filter was applied at 0.5–75 Hz with forward and backward steps, a standardization was performed to calculate exponential moving means and variances, and finally, feature extraction and classification were performed by the deep neural network (DNN).

In this review, it was found that the interfaces in the included studies had between 1 and 64 electrodes for EEG signal acquisition, with 8, 16, and 32 being the most commonly used quantities. The proposal carried out by [35] has highlighted that the system employs a device with only one channel. The Mindwave II device (NeuroSky, San Jose, CA, USA) whose sampling rate is 512 Hz, has a 12-bit resolution. Its supported frequency range is between 0.5 and 100 Hz, and it integrates advanced filters for noise reduction. The proposal was for a BCI system with an experimental model of a 3D robotic hand, which eye blinks were detected based on the electroencephalographic (EEG) signal acquired from the embedded chip of the NeuroSky headset. The authors developed an algorithm in LabVIEW that acquired, processed and implemented the instructions of the robotic hand, and its operation was based on the voluntary blinking of the user’s eyes according to a graphical control panel (one blink enabled the transition between commands for the extension and flexion of the five fingers, and two blinks selected the specific command that triggered the action associated with any given finger). Additionally, the system incorporated modules to achieve integration between the three devices used: the Arduino Uno board, the NeuroSky headset, and the computer.

#### 3.3.2. BCIs Supporting Upper Limb Motor Rehabilitation

As mentioned in Section 3.2.1, the studies were divided according to which limb was being rehabilitated by the BCI system. The percentages indicated that the most studied applications are those involving the upper limb. Similarly, the studies were separated into two groups, taking into account whether the application involves the intention to move the limb or whether it involves assisting with movement.

##### Motor Intention for Upper Limbs

Within the BCI systems presented by each of the included studies, applications divided into the following classes were implemented: virtual limb simulators or avatars, video games, orthoses, and soft exoskeleton. It is important to note that the works involved at least one form of feedback to the user, including haptic stimulation. For example, [48] proposed a motor imagery based BCI for a virtual avatar featuring the upper limbs, which employed functional electrical stimulation (FES) as feedback to provide sensorimotor integration for rehabilitation.

First, to carry out the acquisition of the proposed [37], a recoveriX system (g.tec medical engineering GmbH, Schiedlberg, Austria) and 16-channels with sampling rate of 256 Hz were used. The bandpass filter was set at 0.1 to 30 Hz and also set to extract the features, a fifth-order Butterworth filter with pass frequency from 8 to 30 Hz was employed. Next, the CSP algorithm was used and LDA was utilised as the classification method. The operation of the system consisted of delivering sound and visual signals to the user as an instruction to execute wrist dorsiflexion, while mirroring the avatar’s hand movement. Also, the FES was activated when the system detected the user was imagining movement on the instructed side, in order to help cause the wrist dorsiflexion. According to the participation of 16 people, the results were evaluated by means of the Fugl-Meyer Assessment (FMA) of motor function. As a result, a higher level of improvement was obtained in the patients treated with the BCI-FES system compared to those who did not use it.

Avatars are definitely a promising application to support rehabilitation processes, offering benefits and improvements in performance outcomes [70,71]. For example, one of the papers that integrated EMG signals, although no difference was identified in terms of correlation values between the three experimental conditions (repeating the movement from memory, repeating the movement while viewing it on a screen, and repeating the movement while viewing it in immersive virtual reality), showed that the use of immersive virtual reality resulted in higher alpha desynchronization during movement. In addition, they found that using EEG and EMG together lead to a better result. This development, led by [29], is a virtual avatar presenting either upper limb movements of shoulder flexion-extension, shoulder abduction-adduction, or elbow flexion-extension. For this purpose, the interface used an EEG cap (BioSemi ActiveTwo, Amsterdam, The Netherlands) with 32 active electrodes placed according to the 10–20 system and using a sampling frequency of 2048 Hz (later reduced to 128 Hz). It utilized four surface EMG electrodes (Delsys Trigno Wireless EMG, Natick, MA, USA), with a sampling frequency of 2000 Hz. Subsequently, for EEG signal acquisition, after downsampling the signal from 2048 Hz to 128 Hz, a zero-phase fourth-order Butterworth filter with cutoff frequencies of 0.1 and 2.0 Hz was applied. Then 11 features were selected, and by linear regression, classified. At the same time, seven features were selected from the EMG signals and classified using a 45-neuron network. The second stage of the architecture corresponded to the application of another multiple linear regression model to obtain the final result of the “predictor”. Finally, the prediction entered the virtual scenario where the avatar was floating, developed using Unity 5.3.

Works based on games, as is the case of the proposal by [30], showed effectiveness when using the interactive game with virtual reality, since the performance was higher compared to studies using traditional rehabilitation. However, the authors commented that the clinical experiments only collected data from 12 subjects, so the results may contain errors. The proposed application is a shooting game using virtual reality, an interactive system for upper limb rehabilitation that integrated a motion device (MTD) and measurement of EEG signals to monitor patient treatment in real time. The interface was based on identifying the trajectory of hand movement and the angle of wrist pronation and supination. The system proposed an EEG device whose electrodes were placed according to the 10–20 system and, after the signals were received from the Cz electrode, the theta wave and beta wave were extracted using the Fast Fourier Transform (FFT) and a bandpass filter. Then, the theta-beta ratio (TBR) value was calculated to classify the attention levels, which were compared with the threshold in the virtual environment developed with Unity. In a basic sense, the interface monitored the subject’s attention and used virtual reality to enhance the person’s interest. During treatment, the system collected data on a baseline in order to evaluate the progress and effectiveness of the patient’s rehabilitation. Additionally, it integrated visual and auditory stimuli that are activated when the user’s attention dropped.

Another study that involved using games as BCI applications was the one that [38] presented. They proposed a BCI system integrated with VR and MI to rehabilitate upper limbs. This was the previously mentioned rowing game where the user collected flags. For online EEG data processing, the signals were processed with a bandpass filter for frequencies from 8 to 30 Hz, then a CSP, and finally a LDA sent the signals to a finite state machine (FSM). Also, for the offline EEG analysis, a high pass filter at 1 Hz was used, and then an independent component analysis and Welch’s method were utilized to obtain the power spectral density at the frequencies alpha (8–12 Hz), beta (12–30 Hz), theta (4–7 Hz) and gamma (35–90 Hz).

In terms of hand and wrist rehabilitation, two proposals were identified in the review. The first application is a BCI-controlled electrically-actuated hand orthosis [41], where authors designed an experiment that consisted of voluntarily grasping and relaxing one hand to cause the orthosis placed on the opposite hand to close or open, according to a textual signal displayed on a monitor. Data acquisition was carried out in real time using a 60-channel EEG device located according to the international standard 10-10. However, 32 electrodes were used together with a bio amplifier, a 0.01 Hz bandpass filter with a sampling frequency of 256 Hz using a resolution of 22 bits. Data analysis for offline mode was performed in MatLab using an artifact rejection algorithm, PSD, dimensionality reduction with principal component analysis (CPCA), and its features were extracted using LDA. A Bayesian classifier was then employed and evaluated using cross-validation. Finally, these data were stored for real-time EEG analysis and during online operation, where data was acquired every 0.25s, and they were then combined in a data window. Afterwards, the orthosis system was modeled as a binary state machine and calibrated as a whole. Users demonstrated control of the orthosis after training/calibration, with average performances of 1.15 false alarms and 0.22 omissions per minute. Additionally, the analysis indicated a correlation of 0.58 between voluntary movements mediated by the BCI interface and, based on the results obtained in the online test, cross-correlations indicated that control of the system could be maintained over time and that high correlation coefficients might be achieved.

The second application was aimed to support the hand rehabilitation process developed an interface for use of a soft finger exoskeleton [46]. In the study authors explained that they acquired the data coming from six channels of the Emotiv EEG headset device (San Francisco, CA, USA), a bandpass filter from 4 to 30 Hz, and extracted the features by applying steps such as ERPset energy, differences of ERPset, energy, FilterSet, zero crossing rate, and FFT. Classification was performed in MatLab using learning tools and classifiers such as LDA, QDA, and SVM. The output of this stage was the action input for the control of the virtual actuators and the hand actuators. The result of the project, although it had an important limitation, which was related to a slight delay in signal processing and actuator control (1 second), achieved an overall manual control accuracy rating of 40% and 30% for automatic control in BCI online.

##### Assisting with Movement in Upper Limbs

Similarly, to assist with movement as support in rehabilitation processes, the BCI applications was based on a 3D robotic hand, a mobile robot, a virtual hand simulation and various video games.

The 3D robotic hand was proposed by [35]. As previously mentioned, authors developed a BCI system with an experimental model of a 3D robotic hand. The authors noted that the study produced an efficient model with which training sessions aimed at increasing safety and confidence in BCI interfaces could be offered.

The study proposed by [36] utilised a BCI interface to control a mobile robot based on EEG analysis and two mental tasks: a relaxed state and the motor imagination of the right hand. In the first session, participants received no feedback (it was simply used to set the classifier parameters), and in the second session, they received continuous feedback. The interface employed two channels according to the international 10–20 system, the amplification was carried out by a Guger Technologies (Schiedlberg, Austria) device, and it was digitised at 128 Hz with an NI USB-6210 data acquisition card. Subsequently, the algorithms designed for processing were implemented in MatLab. An LDA classifier was used both for the first session where the error time was calculated with cross-validation and for the second session where the extracted feature parameters were used. The control commands were then translated into four movements: turn 90 degrees to the right, turn 90 degrees to the left, move forward a fixed distance, and move backward a fixed distance. The virtual environment was created using the C programming language with OpenGL for the graphics, OpenAL for the audio, and ODE for the physical simulation. Also, in a second phase, they experimented with a mobile robot (EPFL educational e-puck, Lausanne, Switzerland). At the end of the experiment, an average accuracy of 74.84% was obtained. The authors indicated that it would be interesting to investigate the probability of a navigational command based on the previous sequence of movements in order to organise the commands in order of probability. They also pointed out that the results were less satisfactory for case two, presumably because it was the first time the subjects were confronted with an audio-only interface.

Another proposal that has already been mentioned was made by [39]. Authors designed three experimental conditions in the immersive conditions of multimodal virtual reality with motor priming (VRMP), whose objective was to execute the motor task before training, to have an immersive multimodal virtual reality condition and, to have a control condition with 2D feedback. Using questionnaires, authors collected data on workload, kinesthetic imagery, and presence. Among the findings, they found an increased ability to modulate and enhance brain activity patterns and a strong relationship between electrophysiological data and subjective experience.

In terms of games, the first one identified came from [44]. The game consisted of hitting one of the targets through employing a motor imagination interface and virtual reality. The next was a novel work whose system consisted of six training scenarios: nine training movements that could be chosen by the physician according to the patient’s needs, as well as real-time visual, haptic, and auditory feedback; it was developed by [43]. This MI-BCI and VR-based system to rehabilitate the upper limb function of stroke patients employed 8 channels of a 64-channel EEG device. The VR environment was developed in Unity3D and 3ds Max, which was based on presenting upper limb animations of 3D models.

As in most of the studies analysed in the review, EEG signals were taken from MI, and the processing system was based on the use of MatLab, which performs pre-processing, feature extraction, and classification. The communication between the devices happened through a UDP protocol and the control commands were directed to the 3D model. As a result, motor imagery accuracy increased using visual feedback through the games and the average correct rate for five participants increased by 18.7%. Authors wrote that the VR environment could be designed depending on the group of participants and according to their needs in order to increase rehabilitation performance.

#### 3.3.3. BCIs Supporting Lower Limb Motor Rehabilitation

Although the studies corresponding to lower limb rehabilitation identified for inclusion in the review were the minority, it was also important to categorise them according to the purpose of the application. As a result, four studies fell into the motor intention category and two into the movement assistance category.

##### Motor Intention for Lower Limbs

For articles focused on motor intention, the final identified applications within BCI systems were scenarios with avatars, dorsiflexion exercises with haptic stimulation, and a novel robotic monocycle.

It has been shown that FES technology has produced good results in rehabilitative applications targeting movement restoration [72], and that the combination of FES and VR can be used to reduce difficulties in performing MI tasks and improve classification accuracy. In [33], an interface whose BCI application is a ball kicking scenario based on VR, MI, and FES was proposed to stimulate users’ lower limbs, so that they experience muscle contraction and improve their attention prior to seeing motor imagery. For this purpose, a 32-channel EEG acquisition system (a NeuroScan system including Quick-cap, Grael amplifier and Curry8 software) was used. The electrodes were placed according to the 10-20 system with a sampling frequency of 256 Hz. Then a bandpass filter, a window with a length of 512 sampling points sliding along the time axis at 256-point intervals, the common spatial pattern (CSP) algorithm, a support vector machine (SVM), and the radial basis function (RBF) were all used. The motion scenario, which consisted of kicking the ball, was designed with Unity3D. The results obtained showed that the group subjected to VR and FES obtained the best performance, classification accuracy, and activation intentionality.

Another example of integration with FES systems was developed by [42]. Authors presented a BCI system for direct control of foot dorsiflexion, whose experiment consisted of recording and storing EEG signals in offline mode, and then using these data to classify EEG signals with online mode operation. Signal acquisition was performed using an EEG device with 64 electrodes placed according to the 10–20 system, amplified and bandpass filtered from 0.01 to 50 Hz, a sampling rate of 256 Hz, and a resolution of 22 bits. Dimensionality was reduced by a combination of CPCA and AIDA. Subsequently, the data were sent to a Bayesian linear classifier, and finally, the algorithms were implemented in the BCI software. The FES stimulator that was integrated into the interface was controlled by means of a microcontroller, a finite state machine, and a C-language program. The requests correspond to turning on the stimulator and its intensity, and the instructions to perform the dorsiflexion task were presented as textual signals on the computer screen. As a result, all subjects were able to perform the task without omissions and BCI-FES-mediated foot dorsiflexion epochs were highly correlated with voluntary foot dorsiflexion epochs with latencies ranging from 1.4 s to 3.1 s; also, the correlation coefficient ranged from 0.59 to 0.77, and the classifier performance was 53%. The authors suggested that, with additional modifications, the proposed BCI-FES system may offer a novel and effective therapy in rehabilitation.

Regarding avatars, in the study of [40] a real-time closed-loop BCI system was proposed, which decoded the joint angles of the lower limb from EEG during walking in one, and then controlling the avatar. The system was based on 64 channels (ActiCap, Brain Products GmbH, Gilching, Germany) localised according to the international 10–20 system and a wireless interface to transmit data sampled at 100 Hz. Signal processing was performed using C++ language, and a robust adaptive filter was applied to filter flicker and eye movement. Features were extracted in the 0.1 to 3 Hz band (delta) in order to obtain the joint angle signals for subsequent use of unscented Kalman filter (UKF) as a decoder. To update the UKF parameters, CLDA was applied and the data was collected in a buffer. Finally, to obtain the accuracy levels for decoding, EEG signals were analyzed by standard deviation (STD) and signal to noise ratio (SNR). Likewise, the symmetry ratio (SR) and the ROM of the avatar’s lower limbs were obtained to calculate the gait symmetry and the quality of the avatar’s control. The satisfactory results achieved showed that the average decoding accuracy increased in eight days, the users adapted to the avatar, and the authors noted that the signal stability over the eight days was influenced by states such as attention, motivation and fatigue, suggesting that these states should be quantified in future studies.

Additionally, another novel BCI application was identified using entertainment and/or sport devices. The publication in [45] proposed to use a robotic monocycle, a neurorehabilitation platform based on EEG, surface electromyography (sEMG) and immersive virtual reality (IVR) to move the user’s legs. The system consisted of a customized electronic board to control the monocycle according to the user’s MI, sEMG signals that were collected from the leg muscles in order to identify onset and displacement, a serious game, and the monocycle, which was equipped with inertial sensors placed on the pedals that were used to measure the cadence developed by the user while pedaling. To acquire the EEG signals, a device that recorded 22 channels, a fifth-order Butterworth bandpass filter from 8 to 30 Hz was used, and the CSP, filter bank (FBCSP), and Riemannian kernel (RK) methods were used for feature extraction. Subsequently, the interface integrated the LDA and the RDA classifiers to recognize the resting and moving states of the feet. Also, the game was designed using Unity 3D Personal Edition, which was run on a Windows computer. The development was able to recognize MI patterns from foot motion with an average accuracy of over 80%. The authors also indicated that the LDA classifier may be suitable for online implementation, which decreases the cost of calculation in the calibration and validation stages.

##### Assist with Movement in Lower Limbs

Wheelchairs have been an important part of BCI applications, as they are widely used and have been under different scientific and technological fields that seek their complete evolution. The two studies included in the review were focused on wheelchairs. However, one of the studies was based on controlling a virtual wheelchair, while the other was based on controlling both a virtual and a real wheelchair.

On the one hand, Ref. [31] posed a paradigm for controlling a 2D virtual wheelchair. The paradigm was based on multi-class discrimination of the spatially distinguishable phenomena of event-related desynchronization and synchronization (ERD/ERS) in EEG signals associated with motor execution and hand MI. A 27-channel device with a sampling rate of 256 Hz, placed in an elastic cap according to the international 10–20 system, was used. The signals coming from the channels were amplified and filtered within 0.1 Hz and 100 Hz. To extract the features, the SLD was applied as a spatial filter, the temporal filtering was performed by power spectral density estimation, and then an MLD was applied. Classification to obtain control signals was performed in two steps: the first was to identify whether the signal belonged to control commands (true positive or TP) or non-control commands (true negative or TN) and associated with motor tasks and EEG patterns. Finally, the control command was sent discretely while the wheelchair was continuously moving. The study showed high accuracy, as the incorporation of ERS into the paradigm improved the spatiotemporal feature contrast of ERS vs. ERD, the average target hit rate was 98.4% with MI, and the average time to reach a target at 10m distance was approximately 59 s.

Also, Ref. [32] proposed a hybrid BCI based on MI and P300 potential to control a wheelchair, both virtual and real, in a study where participants were able to effectively control the direction and speed of the wheelchair. A 15-channel device was used to acquire EEG signals, which were amplified, sampled at 250 Hz, and filtered between 0.5 and 100 Hz with a band-pass filter. They were then spatially filtered with CAR, then with a band-pass filter at 8–32 Hz and. Spatial patterns were calculated using the one vs. the rest common spatial patterns (OVR-CSP) method. LDA was used, and finally, by using MI, hand movement was associated with the wheelchair’s turning direction. For deceleration, MI of the foot was used, and for acceleration, attention to a blinking button on the graphical user interface (GUI) was used.

#### 3.3.4. BCIs Supporting Upper and Lower Limb Motor Rehabilitation

One of the 18 studies involved the support of upper and lower limb rehabilitation. The interface, a work developed by [34], proposed a multi-class system based on the modulation of sensorimotor oscillations with MI, evaluated in two studies. The first one corresponded to training users to imagine hand and foot movement in response to visual cues, and the second one to evaluate the information transfer rate (ITR) of the interface in an asynchronous application, corresponding to navigation in a two-dimensional maze. The interface design offered three modes of operation: asynchronous, user-dependent automatic parameter selection based on initial calibration, and incremental update of the feedback data classifier parameters. The acquisition of EEG signals was based on the use of 16 electrodes placed according to the 10–10 system. Afterwards, they were spatially filtered for further feature extraction, which relied on the estimation of the calculated spectral power in individualised frequency bands, which were automatically identified by an AR-based model. MI classification was performed by means of a multinomial logistic regressive classifier.

#### 3.3.5. User Feedback within the BCI Systems

After the application control system receives signals and uses them to run an application, it integrates a final stage called feedback. This is a really important part of the process, and most of the current BCI applications incorporate it. The feedback given to the user can be visual, auditory, or haptic. All three kinds have demonstrated that they can enhance motivation and minimize frustration in training, rehabilitation processes, and using assistive devices [73,74].

As another important point, it was observed that the most commonly used technology for the design of virtual environments as part of the visual feedback was Unity, a multi-platform, a fully integrated professional game engine for interactive content such as creating games and visualization, etc. [75]. Six of the 18 studies based their virtual environment on Unity, which suggests that this tool is very useful in the design of brain-computer interfaces.

Likewise, the Oculus Rift virtual reality system, which is a line of virtual reality headsets developed and manufactured by Oculus VR (Menlo Park, CA, USA) [76], was the most widely used for fully immersive virtual reality applications. According to the results presented in [38], whose proposal integrated the Oculus Rift system and Unity, it was noted that although the overall classification performance remained low, the results showed an increase in brain activation due to the effect of virtual reality training and feedback. In addition, the authors found improvements in upper limb scores and increases in brain activation measured by fMRI, indicating neuroplastic changes in the brain’s motor networks.

The application in [38] consisted of and examined the effectiveness of a BCI system integrated with virtual reality and motor imagery to rehabilitate upper limbs. The objective of the study was to row a boat through graphics in order to collect the most flags in a fixed period of time. The interface used the Enobio 8 sensor with eight EEG channels to acquire 24-bit data at 500 Hz, according to the 10–20 system. Vibrotactile feedback was delivered using a custom module made with an Arduino Mega, board motors, and vibrating parts. Auditory feedback included ambient sounds, paddle movement, and events for when the player captured a flag.

Another project presented in [39] which also employed the Oculus Rift system and whose visual and auditory feedback was designed with Unity 3D, presented the use of headphones for spatial sound, multimodal VR simulations, and motor priming (MP) in a BCI task with upper limb MI, in addition to proposing a BCI paradigm for patients with low-level motor control.

For this purpose, the authors used 8 active electrodes, a biosignal amplifier, a 256 Hz A/D converter and the distribution according to the 10–20 system. The Leap Motion controller was used to map the movement of the hands and the OpenVibe platform was used for signal acquisition and processing. Feature extraction was given using a CSP filter. Subsequently, for the classification of the left and right hands, LDA analysis was used and then the information was transmitted to the RehabNet control panel (RehabNetCP) through the VRPN protocol, in order to control the virtual environment.

In addition, the type of virtual reality, primarily as part of visual and auditory feedback, was considered with great interest in the systematic review. It was found that, although the majority of the systems featured non-immersive reality, the next percentage presented applications of fully immersive reality.

##### Use of Fully Immersive Reality

The applications within immersive reality are related to avatars and video games. In the study presented in [29], authors found that most of the people involved showed more comfort with this technology compared to the other conditions, which increases the feasibility of further exploring this technology. Authors developed a virtual avatar’s upper limb movements of shoulder flexion-extension, shoulder abduction-adduction or elbow flexion-extension in three conditions, and whose virtual scenario was developed using Unity 5.3.

There were three other fully immersive studies. One was proposed in [38] (system based on a rowing game to collect flags), whose results showed an increase in brain activation due to the effect of virtual reality training and feedback. The second work presented in [39], involved a motor task that was experienced before training, in an immersive multimodal virtual reality condition and in a control condition with 2D feedback, whose analysis indicates that the activation of the ipsilateral primary sensorimotor cortex (SMC) in the mirror neuron system are important in action execution and imitation enhanced by VR. Finally, the third study developed a target shooting game. The authors note that one of the participants stated, “It’s a bit weird, because it feels like my hands.” [44].

##### Use of Semi-Immersive Reality

The studies that feature semi-immersive reality were: the ball-striking based proposal designed in Unity3D [33], the 3D robotic hand by [35] employing algorithms in Labview, and the system featuring upper limb animations in 3D models, developed in Unity3D and 3ds Max, for which it was noted that motor imagery accuracy increased using visual feedback through gaming and that the average correct rate for 5 participants increased by 18.7%.

##### Use of Non-Immersive Reality

Finally, obtaining the highest percentage, there were 11 studies involving non-immersive virtual reality. There were the following: a game proposed in [30], which showed greater effectiveness compared to traditional rehabilitation, a wheelchair [31], the system to control a mobile robot based on EEG analysis, and two mental tasks (relaxed state and motor imagination of the right hand) [36], systems with FES [37,42], the system that decoded lower limb joint angles during walking [40], the rehabilitation hand [46] and the robotic monocycle of [45].

Also, Ref. [34] proposed a multi-class interface based on the modulation of sensorimotor oscillations with motor imagery, which was evaluated in two studies and, whose feature selection was carried out by a criterion based on mutual information. The results showed an accuracy of 74.84%, thus demonstrating that the use of interfaces outside the laboratory is possible.

## 4. Discussion

In recent years, the great potential of using EEG signals in BCI systems to support the rehabilitation process of human limbs has been demonstrated in proposals such as those evidenced in the systematic review. The evidence shows that the use of EEG signals within a BCI system is optimal and suitable when said system has the following elements: the incorporation of non-invasive and inexpensive equipment, good resolution, ease of use, and portability [77,78,79]. Also, the use of these signals for the development of BCI systems that support rehabilitation processes is natural and intuitive [80] since the process directly extracts information about the user’s motor intention. The use of EEG signals in these systems has received satisfactory results both in terms of system efficiency and patient satisfaction [44]. For this reason, the use of EEG signals was one of the main inclusion criteria, and from the analysis of the studies reviewed it was observed that BCI-EEG systems have a clear validation of their value in real life in terms of efficacy, practicality, and their impact on the quality of life of their users. It is also important to note that future BCI systems should be even more comfortable, more convenient, easier to set up, operate for longer periods of time in all environments, and more easily interact with a wide range of applications through virtual reality [81,82,83,84].

On the other hand, as observed in studies and papers presented in [3,12,85,86], the obstacles when designing and implementing BCI systems that should be taken into account during the development of applications are related to: neurological issues, technological difficulties, ethical concerns, non-stationarity of data from the same user (changes in signal patterns over time), signal acquisition hardware, information transfer rate, and the training process: indeed, longer training should help subjects learn to generate more consistent and distinct MI activity. Additionally, shorter trials and improvements in feedback to the user are also helpful.

Furthermore, in the literature, improvements in rehabilitation processes were identified when feedback elements were integrated in some of its modalities (visual, auditory, or haptic) and virtual environments. For instance, in [36], a higher level of improvement was obtained in the patients treated with the BCI-FES system compared to those who did not use it. It was also shown in [29] that the use of immersive virtual reality resulted in higher alpha desynchronization during movement. The same could be said about the case of the proposal by [30], which showed effectiveness when using an interactive game with virtual reality; the performance was higher compared to studies using traditional rehabilitation. Furthermore, in [35], it was noted that the accuracy of motor imagery increased by using visual feedback through gaming and that the average rate of correctness increased by 18.7%.

In this sense, it became evident that it is particularly essential to provide feedback to the person about the motor intention that has been recognised by the system in order to let the user know if he/she has correctly executed the mental task, which will consequently help the user to better control his/her brain activity and obtain greater efficiency in the rehabilitation process. Indeed, having a feedback opens the possibility to exploit the control of interfaces and even generate personalised models as indicated in [42,44].

Virtual environments in this framework can play a fundamental role. This is because they are useful as a diagnostic and/or rehabilitation tool, and their effects can be monitored by recording both clinical and electroencephalographic data. For example, in [87] the case of a 17-year-old patient is presented. She had a disorder of consciousness (DoC), and a VR training to improve her cognitive-behavioral outcomes which were assessed using clinical scales was presented. At the end of the training, significant improvements in clinical and neurophysiological outcomes were achieved. As a result, it is suggested that neurophysiological data and BCI systems in combination with virtual reality could be used to evaluate the reactions induced by different paradigms and help in diagnosis and rehabilitation.

Although it was evidenced during the analysis of the articles that several strategies have been investigated, in strategies that included motor training, direct observation, and VR techniques, the feedback to the user regarding the use of virtual environments showed more benefits, as users acquired greater confidence and control of the system. On the other hand, as mentioned in [38], even though growing evidence of the positive impact of methods using VR has been demonstrated, patients with low level of motor control have benefited from BCIs. For this reason, it is also important to opt for the use of motor imagination, other feedback methods, or integration with VR. There are people who have severe impairments in their limbs and therefore may not have autonomy to carry out activities of daily living.

Advancements in the DL field have also improved efficiency in the accuracy of processing signals and BCI system performance, as shown in the included paper written by [44], and in another example from [88]. In this study, DL was used in order to recognize four different movements from a recorded EEG. Data from 10 volunteers showed a success rate of 70% from the raw EEG and a success rate of 96% from the spectrum. DL methods are better than machine learning algorithms when the data is complex, unstructured, abundant, and feature rich [89,90]. Also, DL can easily describe complex relationships and preserve the information extracted from brain networks [91] or even as [92] expressed, DL techniques are useful to infer information about the correctness of action in BCI applications.

In addition to observing that training with feedback improved test’s scores, it was evidenced that the incorporation of VR in BCI systems can offer forms of low-cost support, accessibility, ease of use, and agility in reconfiguration [40]. Also, it is important that the variation between calibration, training, and real-time operation continues to be improved in order to obtain high performance percentages and low effects on users’ mental states. Taking into account that studies have confirmed that using mechanisms such us the use of VR promote brain plasticity, produce positive effects in the restoration of motor function, and strengthen rehabilitation processes in human limbs [93,94]. In conclusion, machine learning and deep learning algorithms to classify EEG signals, visual, auditory, and haptic feedback, and EEG within brain-computer interfaces offer wide possibilities of development that efficiently support limb rehabilitation, helping not only the users, but also their families and the people around them to benefit, improving the quality of life of all, impacting social growth.

## 5. Conclusions

Especially for people with motor limitations, a BCI is a system that provides a way to re-establish direct communication with what the user wants to do, making it possible to assist or restore their autonomous mobility [13,15]. This review included papers that focused on supporting limbs rehabilitation through the use of EEG and feedback. The analysed articles demonstrated that the integration of BCIs with visual, auditory and/or haptic stimulation is useful and increases the efficiency of the interface in rehabilitation, since it enhances the physiological and emotional effects of the person.

Inspecting the results and advances in signal acquisition and processing technologies opens the possibility of continuing to innovate the use of this technology as support in motor rehabilitation processes. These systems in combination with non-invasive methods lead to more efficient, economical, fast, and risk-free rehabilitation processes. On the other hand, it was identified that the use of processing techniques, statistics, machine learning, and deep learning are vital in the processing of recent and future works, since they attempt to minimise errors and increase the efficiency and speed with which the system operates.

In general, the studies were based on applications to rehabilitate the upper limbs. The highest percentage of studies were focused on BCI applications related to avatars and video games, so the feedback to the user was mostly visual. However, the use of visual, auditory, and haptic feedback was considerable. It is also important to note that within EEG signal processing techniques, common spatial patterns (CSP) is used together with linear discriminant analysis (LDA) as classification algorithm. On the other hand, the most commonly used type of VR is non-immersive, and importantly, among the studies included according to the inclusion and exclusion criteria, none used augmented virtual reality.

Finally, it was determined that within the systems to support the rehabilitation of human limbs, BCIs offer extensive benefits along with the use of EEG signals and feedback provided to the user. Benefits in training, monitoring, calibration, and motivation may lead to inherent improvements within the rehabilitation processes, the quality of life for people who suffer from motor limitations and will ultimately result in a positive impact on their families and society.

## Figures and Tables

**Table 1:** Inclusion and exclusion criteria.

Inclusion Criteria	Exclusion Criteria
Use of encephalographic signals (EEG)	Application or device developed for rehabilitation in upper and lower limb of the human body unspecified
Applications for the rehabilitation of upper and lower limbs of the human body.	Articles that present analyses of the BCI interface design procedure or are review articles
Applications whose focus is to rehabilitate motor intention or assist with movement	Articles describing brain-computer interfaces with invasive procedures
Use of virtual environments as a feedback technique.	Articles describing brain-computer interfaces with invasive procedures or not indicating EEG signal processing techniques

**Table 2:** Main characteristics of the included studies.

No	Limb Being Rehabilitated	Purpose of BCI Application	Category of BCI Application	BCI Application	Type of Feedback to Uset	Type of Virtual Reality	Reference
1	Upper limb	Motor intention	Virtual limb	Virtual upper limb	Visual	Fully Immersive Virtual reality	[29]
2	Upper limb	Motor intention	Video game	VR shooting game	Visual, Auditory	Fully Immersive Virtual reality	[30]
3	Lower limb	Assist with movement	Wheelchair	2D virtual wheelchair	Visual, Auditory	Non-immersive virtual reality	[31]
4	Lower limb	Assist with movement	Wheelchair	Simulated or real wheelchair.	Visual	Non-immersive virtual reality	[32]
5	Lower limb	Motor intention	Virtual limb	Ball-kicking simulation	Visual, Haptic	Semi-immersive virtual reality	[33]
6	Upper and lower limb	Assist with movement	Video game	Virtual maze	Visual	Non-immersive virtual reality	[34]
7	Upper limb	Assist with movement	Virtual limb	3D robotic hand	Visual	Semi-immersive virtual reality	[35]
8	Upper limb	Assist with movement	Virtual limb	Robot (virtual robot and mobile robot)	Visual, Auditory	Non-immersive virtual reality	[36]
9	Upper limb	Motor intention	Virtual limb	Upper virtual limb	Visual, Auditory, Haptic	Non-immersive virtual reality	[37]
10	Upper limb	Motor intention	Video game	Neurogame (Rowing game)	Visual, Auditory	Fully Immersive Virtual reality	[38]
11	Upper limb	Assist with movement	Virtual limb	Virtual hands through MI.	Visual, Auditory	Fully Immersive Virtual reality	[39]
12	Lower limb	Motor intention	Virtual limb	Avatar walking in a virtual environment	Visual	Non-immersive virtual reality	[40]
13	Upper limb	Motor intention	Orthosis	Electric-action hand orthosis	Visual	Non-immersive virtual reality	[41]
14	Lower limb	Motor intention	Electrical stimulation (FES) system	Dorsiflexion of the foot with a FES system	Visual, Haptic	Non-immersive virtual reality	[42]
15	Upper limb	Assist with movement	Video game	Nine training movements	Visual, Auditory, Haptic	Semi-immersive virtual reality	[43]
16	Upper limb	Assist with movement	Video game	Target shooting game	Visual	Fully Immersive Virtual reality	[44]
17	Lower limb	Motor intention	Virtual limb	Robotic monocycle	Visual	Non-immersive virtual reality	[45]
18	Upper limb	Motor intention	Soft Exoskeleton	A soft finger exoskeleton	Visual, Auditory	Non-immersive virtual reality	[46]

## Data Availability

Not applicable.
